# Mobilization of Regulatory T Cells in Response to Carotid Injury Does Not Influence Subsequent Neointima Formation

**DOI:** 10.1371/journal.pone.0051556

**Published:** 2012-12-11

**Authors:** Amit Saxena, Harry Björkbacka, Åsa Ström, Sara Rattik, Katarina E. Berg, Maria F. Gomez, Gunilla Nordin Fredrikson, Jan Nilsson, Anna Hultgårdh-Nilsson

**Affiliations:** 1 Department of Clinical Sciences Malmö, Lund University, Lund, Sweden; 2 Department of Experimental Medicine, Lund University, Lund, Sweden; 3 Department of Health and Society, Malmö University, Malmö, Sweden; King’s College London, University of London, United Kingdom

## Abstract

**Aim:**

T cells have been attributed an important role in modulating repair responses following vascular injury. The aim of this study was to investigate the role of different T cell subsets in this context.

**Methods and Results:**

A non-obstructive collar was introduced to inflict carotid artery injury in mice and subsequent activation of immune cells in draining lymph nodes and spleen were studied by flow cytometry. Carotid artery injury of wild type mice was associated with mobilization of both Th1 type CD4^+^IFNγ^+^ and regulatory CD4^+^CD25^+^FoxP3^+^ T cells in draining lymph nodes. Studies using FoxP3-green fluorescent protein (GFP) transgenic C57/Bl6 mice demonstrated scattered presence of regulatory T cells in the adventitial tissue of injured arteries as well as a massive emigration of regulatory T cells from the spleen in response to carotid injury. However, deletion of antigen presentation to CD4+ T cells (H2^0^ mice), as well as deletion of regulatory T cells (through treatment with blocking anti-CD25 antibodies), did not affect neointima formation. Also deletion of antigen presentation to CD8^+^ T cells (Tap1^0^ mice) was without effect on carotid collar-induced neointima formation.

**Conclusion:**

The results demonstrate that carotid artery injury is associated with mobilization of regulatory T cells. Depletion of regulatory T cells does not, however, influence the subsequent repair processes leading to the formation of a neointima. The results also demonstrate that lack of CD8^+^ T cells does not influence neointima formation in presence of functional CD4^+^ T cells and B cells.

## Introduction

Vascular repair responses activated by chronic or acute injury play important roles in the formation of atherosclerotic plaques as well as in plaque healing and development of restenosis after angioplasty [1]. These healing responses may be beneficial by promoting plaque stabilization but can, if poorly controlled, also lead to the development of flow-limiting stenosis. Vascular repair responses are primarily regulated by the release of growth factors, but it has also been found that these processes are regulated by both innate and adaptive immune responses [2]–[5]. Experimental models based on catheter-induced injury of rat carotid arteries and peri-adventitial collar-induced injury of mouse carotid arteries have been developed to study neointima formation in response to injury under controlled conditions [6]. Pro-inflammatory innate immune responses, including IL-1 and Toll-like receptor activation, have been shown to promote neo-intimal growth [4], [7], and several studies have attributed an important role of chemokines and adhesion molecules in this process [8]–[10]. However, the role of adaptive immunity in regulating vascular repair responses appears to be much more complex. Carotid injury of mice deficient for CD1d, a MHC class I-related molecule required for presentation of lipid antigens to NKT cells, is associated with reduced neointima development [11]. In contrast, Rag-1^−/−^ mice, which lack mature T and B cells, are characterized by enhanced neointima formation following arterial injury [12] suggesting that adaptive immune responses also serves to control the extent of injury-induced repair processes. In accordance with this notion, T cell depletion has been found to result in increased neointima formation following balloon catheter-injury of rat carotid arteries [3] and T cell transfer into Rag-1 mice reduces neointima formation down to similar levels as in wild-type mice [13]. Recent studies by Dimayuga and coworkers demonstrated presence of activated CD4^+^ and CD8^+^ T cells in draining lymph nodes one week after arterial injury and showed that transfer of CD8^+^, but not CD4^+^, T cells reduced neointima formation in Rag-1 mice [14]. The ability of CD8^+^ T cells to inhibit neointima formation was associated with a cytotoxic activity against smooth muscle cells suggesting that the effect of CD8^+^ T cells was mediated through cytolysis of neointimal smooth muscle cells. Although these findings argue against a role for CD4+ T cells in modulation of vascular repair responses, previous studies have shown that the Th1 cytokine interferon (IFN)γ has a bimodal role following vascular injury inhibiting the earliest stages of neointima formation while promoting this process at later stages [13]. Activation of naïve CD4^+^ T results in differentiation into different subsets with partly opposite functions, including pro-inflammatory Th1 cells, Th2 cells that mediate antibody isotype switch in B cells and suppressive, anti-inflammatory regulatory T cells (Tregs). Accordingly, it cannot be excluded that the CD4^+^ T cell population contains subsets of cells with different effect on neointima formation. In the present study we assessed mobilization of different subtypes of CD4^+^ T cells in draining lymph nodes following carotid injury of wild type mice. We also assessed the effect of inhibiting antigen presentation through MHC class I and II molecules as well as the effect of removal of regulatory T cells on neointima formation after injury.

## Materials and Methods

### Animals

The study was approved by the Lund/Malmö ethical committee and conformed to the guide and use of laboratory animals published by the US National Institutes of Health (NIH publication No. 85-23, revised 1996). Mice with a targeted deletion in the *Tap1* gene (Tap1^0^; B6.129S2-*Tap1^tm1Arp^*/J, stock number 002944), which are defective in the stable assembly, intracellular transport and surface expression of MHC class I molecules, mice deficient of the MHC class II genes H2-Ab1, H2-Aa, H2-Eb1, H2-Eb2 and H2-Ea (H2^0^; B6.129S2-*H2^dlAb1-Ea^*/J, stock number 003584), and Foxp3-green fluorescent protein (GFP) transgenic C57BL/6 mice that co-express GFP and the regulatory T cell-specific transcription factor Foxp3 under the control of the endogenous promoter (B6.Cg-*Foxp3^tm2Tch^*/J, stock number 006772) were purchased from Jackson Laboratory. Wild type (WT) mice C57/Bl6 mice were purchased from Taconic. The animals were fed chow diet and given water ad libitum.

**Figure 1:**
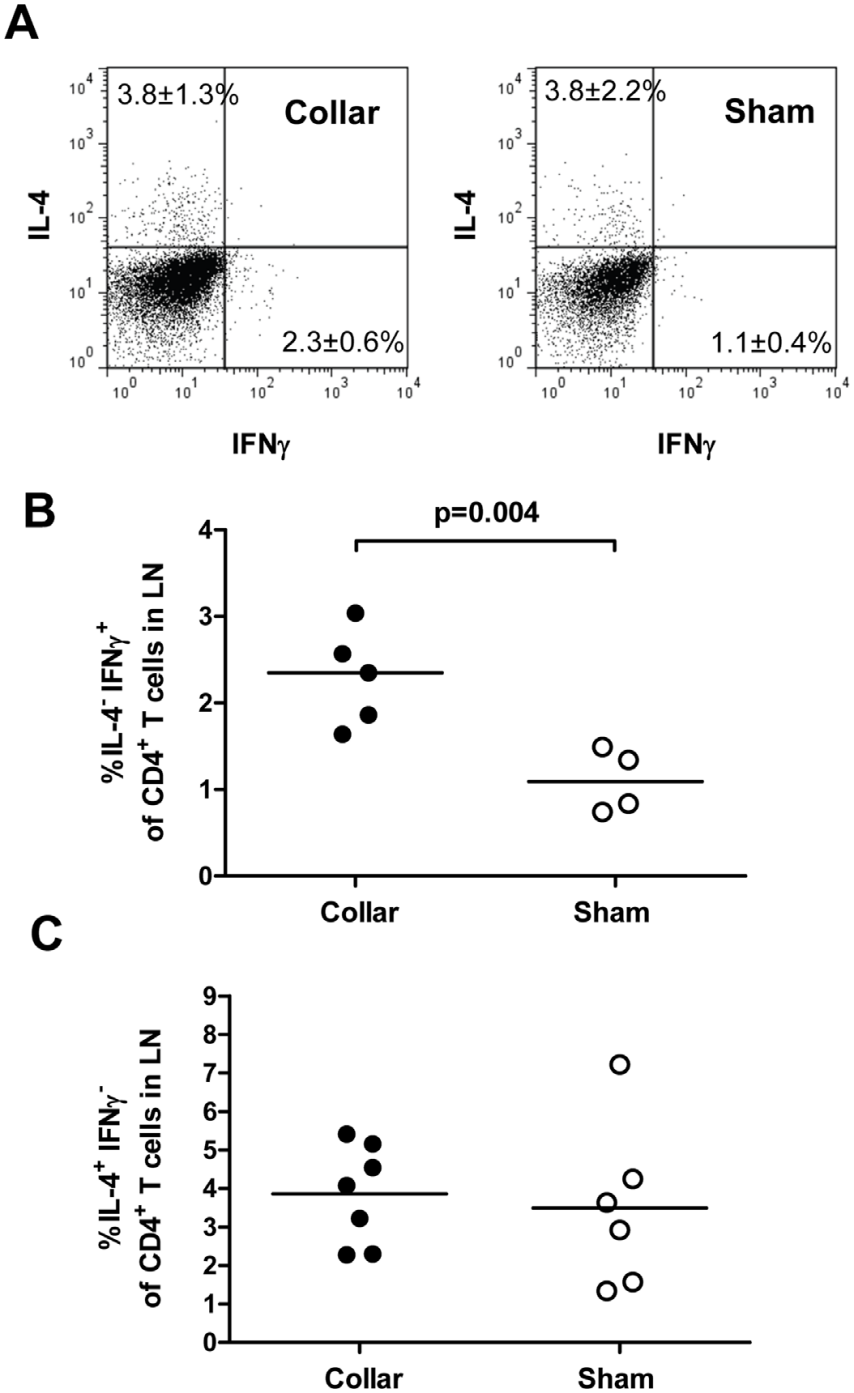
Increased IFNγ producing T cells in draining lymph nodes after injury of the carotid artery. Cells were isolated from pooled lymph nodes (LN) of injured or sham operated mice (day 3), CD4, IFNγ and IL-4 and analyzed by flow cytometry. A. Representative dot plots. B. IFNγ^+^ cells as a percentage of CD3^+^CD4^+^ T cells in draining lymph nodes. C. IL-4^+^ cells as a percentage of CD3^+^CD4^+^ T cells in draining lymph nodes.

**Figure 2:**
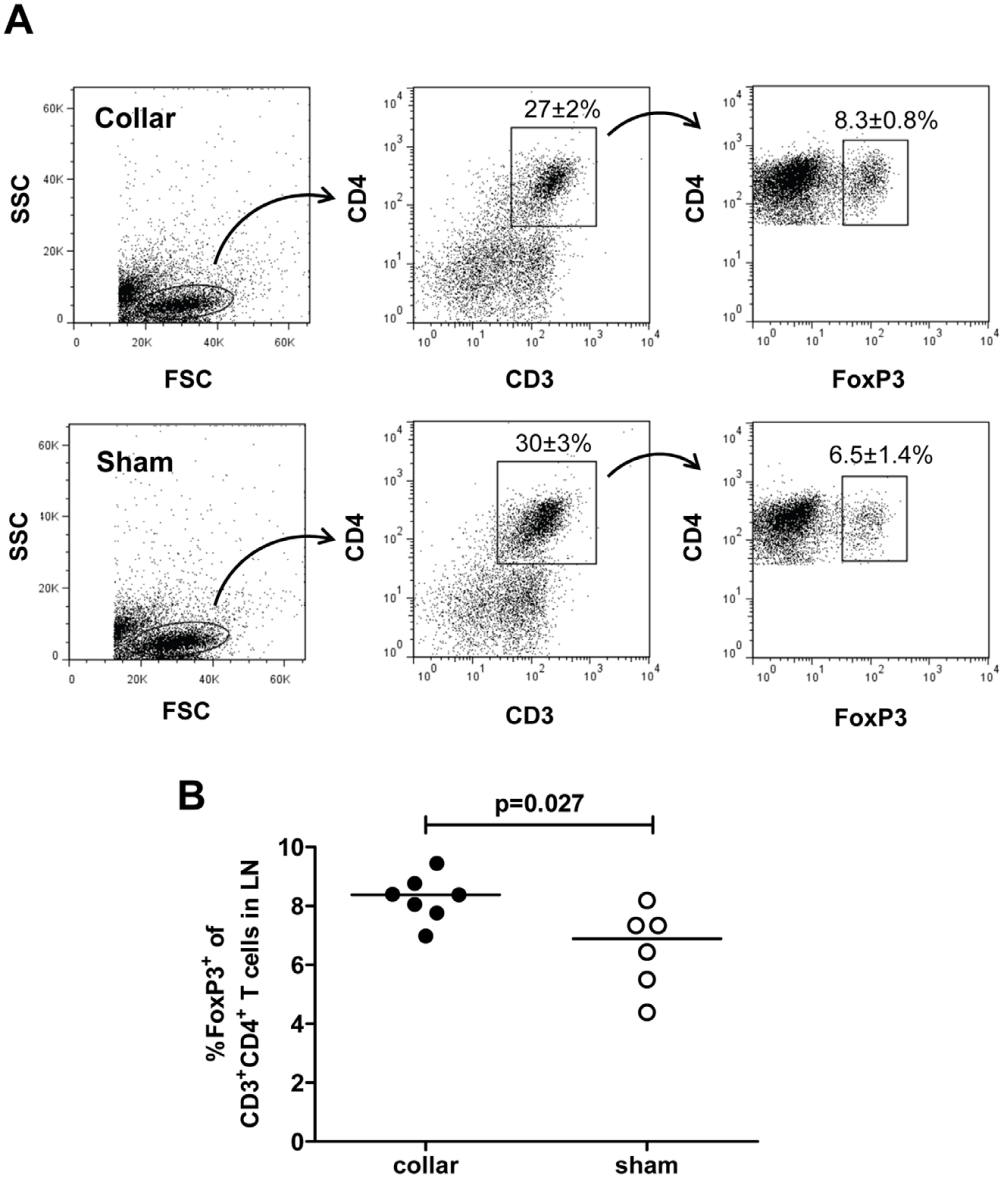
Increased regulatory T cells in draining lymph nodes after injury of the carotid artery. Cells were isolated from pooled lymph nodes (LN) of injured or sham-operated mice, stained with antibodies against CD3, CD4 and FoxP3 and analyzed by flow cytometry. A. Representative dot plots. B. FoxP3^+^ cells as a percentage of CD3^+^CD4^+^ T cells.

**Figure 3:**
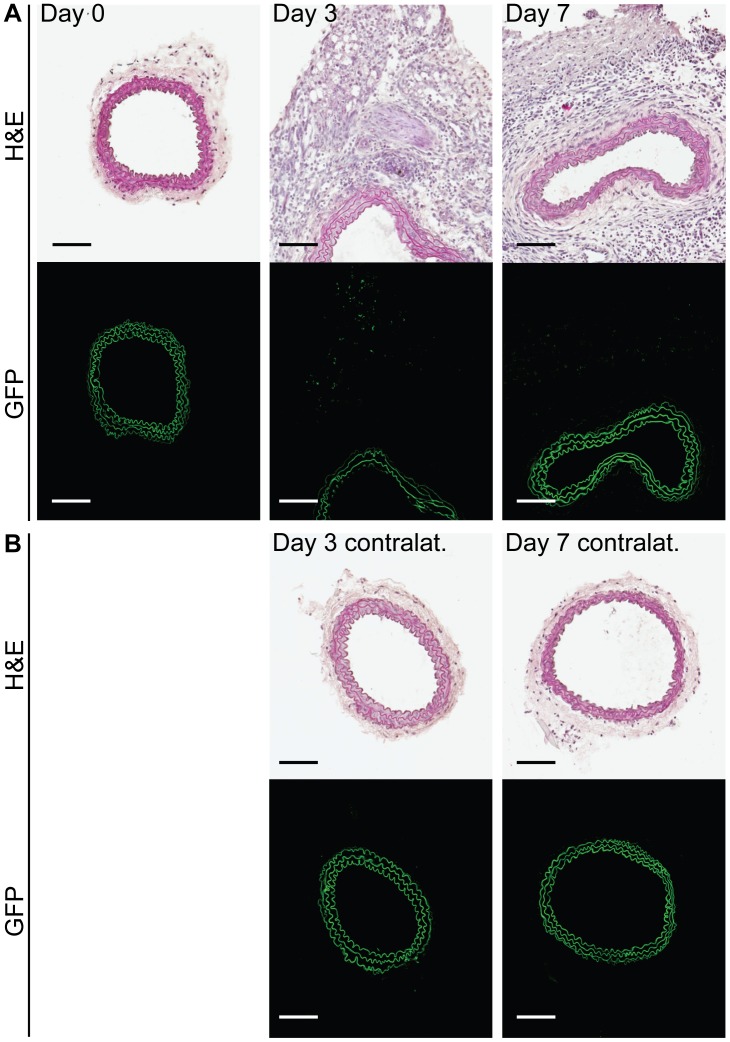
Regulatory T cells do not accumulate in the injured artery but are observed in the periadventitial tissue after surgery. A. Representative sections of carotid arteries from non-operated control mice (Day 0), and from mice 3 or 7 days after injury showing the presence of FoxP3-GFP^+^ cells (green scatter) in the adventitial granulation tissue. Upper row shows sections stained with Harris hematoxylin (H&E); lower row shows confocal images of the same arteries (consecutive sections). B. Corresponding images for the contralateral arteries of the mice shown in A. Autofluorescence from the elastic laminae in the arterial wall is shown in green. Scale bars = 100 µm, n = 5 mice per group.

**Figure 4:**
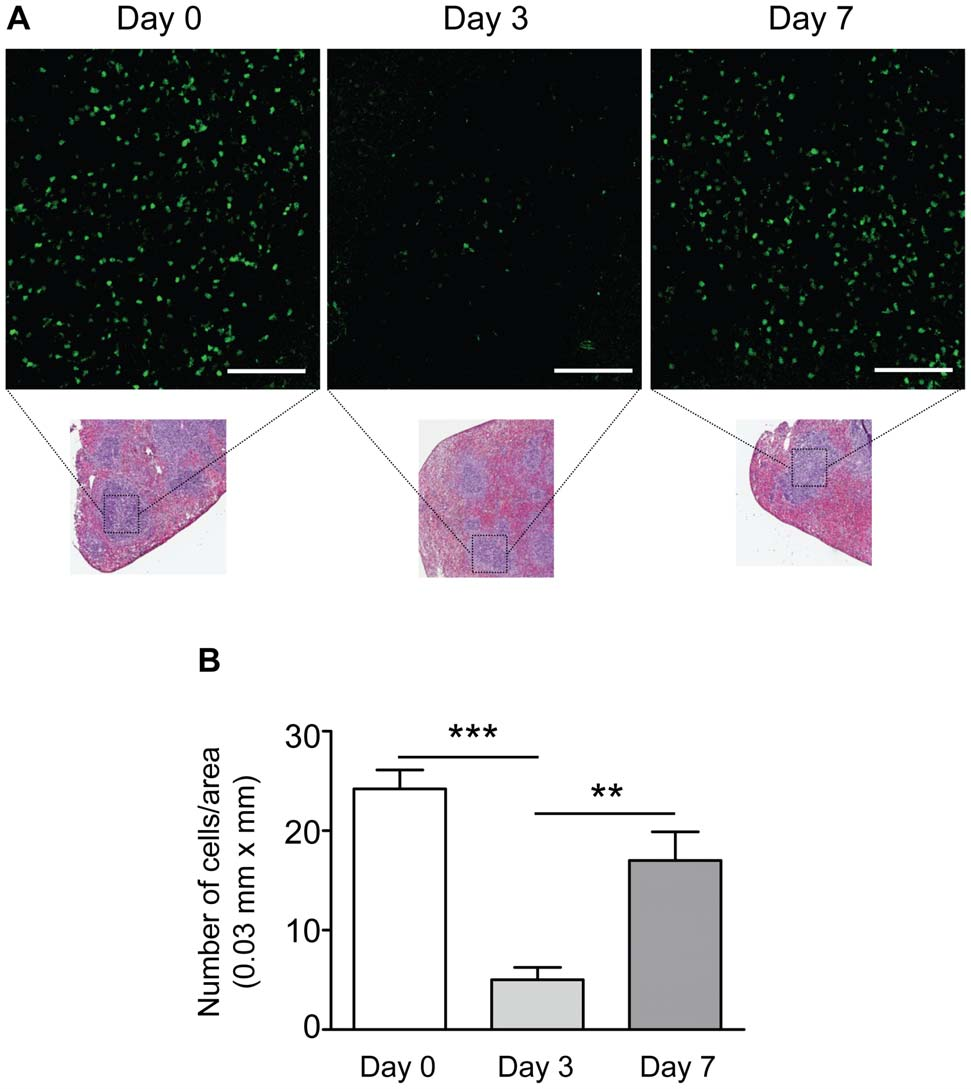
Regulatory T cells emigrate from the spleen after injury of the carotid artery. A. Representative confocal images showing FoxP3-GFP^+^ cells (green) in the spleen of non-operated control mice (Day 0) and in the spleens of mice 3 or 7 days after injury. Smaller insets show spleen sections from the same mice stained for H&E for visualization of tissue architecture and the white pulp areas that were imaged. Scale bars = 100 µm. B. Summarized data from confocal experiments showing significantly reduced number of FoxP3-GFP^+^ cells in spleens 3 days after injury and restored levels at day 7. N = 5 mice per group; ***P < 0.001, **P < 0.01.

**Figure 5:**
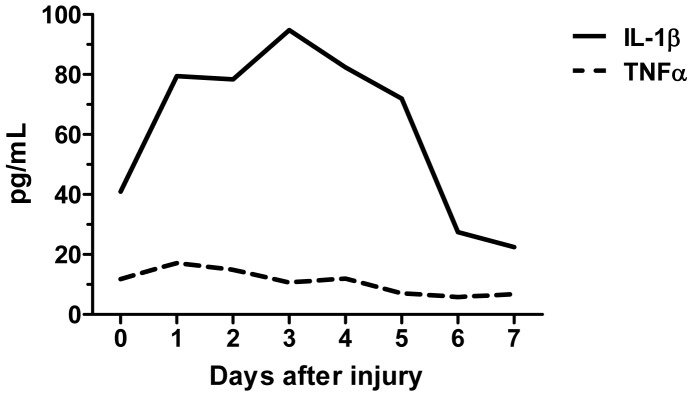
Increased systemic inflammatory response after injury of the carotid artery. Plasma levels of IL-1β and TNFα measured with Luminex every day (day 0 through 7) after surgical placement of a carotid collar to induce vascular injury. The average level in two mice per time point is shown.

### Periadventitial Collar Injury

At approximately 16–18 weeks female Tap1^0^, H2^0^ mice, WT mice (C57/Bl6) and FoxP3-green GFP transgenic C57/Bl6 mice were anaesthetized using (ketamine/xylazine). The use of anesthetics and the introduction of a non-occlusive plastic collar have been previously described in detail [4]. Mice were sacrificed after 3 or 21 days and carotid arteries (both injured and contralateral) processed for histopathology. FoxP3-GFP transgenic C57/Bl6 mice were sacrificed 3 or 7 days after surgery and spleens and carotid arteries (both injured and contralateral) were processed for detection of GFP positive cells by confocal microscopy or for histology (Harris hematoxylin). Non-operated FoxP3-green fluorescent protein (GFP) transgenic C57/Bl6 mice were used as controls (day 0). Carotid arteries were perfusion fixed with Histochoice (Amresco), dissected out and stored in Histochoice (Amresco), at 4°C until analysis. For depletion of regulatory T cells mice were given an intra-peritoneal injection with 100 µg of purified IgG1 κ isotype control antibody or 100 µg purified anti-mouse CD25 (clone PC61) antibodies (Biolegend, San Diego, CA, USA) 2 days before the collar placement. A second dose of the antibody was given 7 days later.

**Figure 6:**
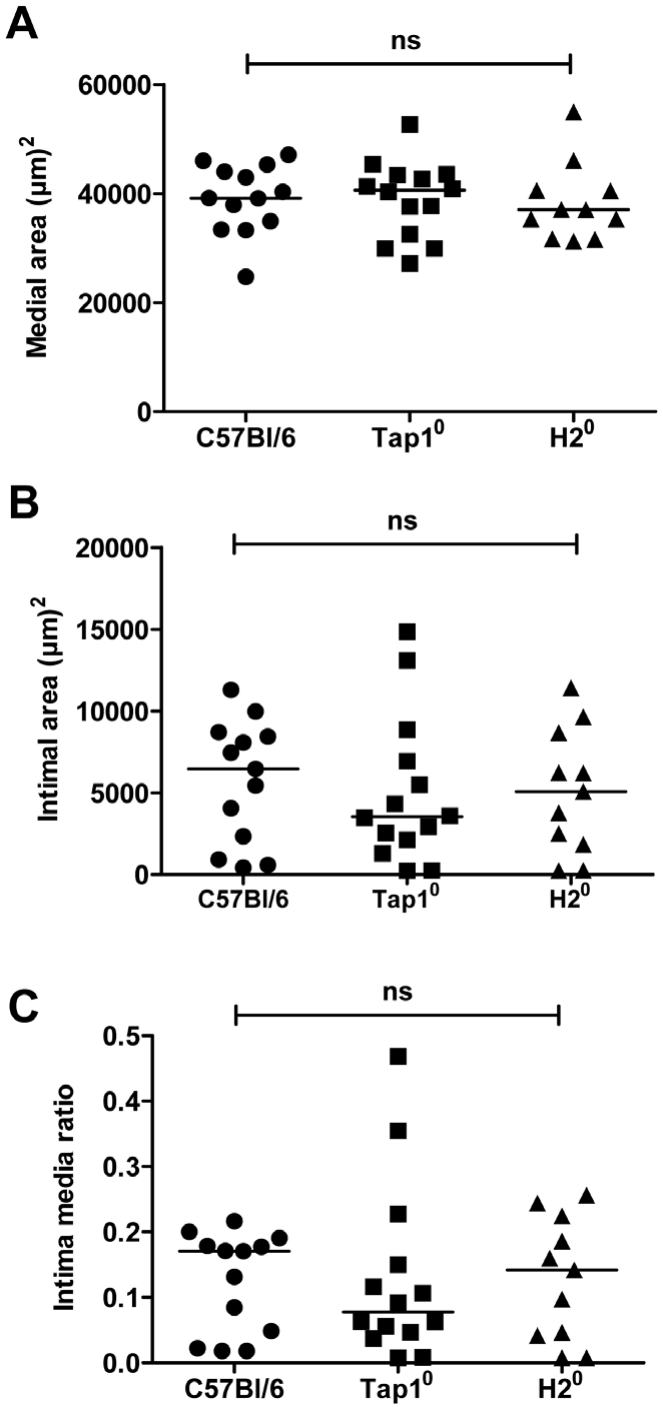
MHCI or MHCII deficiency does not alter the vascular response to injury. Morphometric measurements of carotid artery sections after vascular injury (day 21) in C57Bl/6 mice, Tap1^0^ mice (lacking MHC class I expression) and H2^0^ mice (lacking MHC class II expression). A. Medial area. B. Intimal area. C. Intima-media ratio.

### Morphometric Analysis and Confocal Microscopy

Preparation/fixation of the serial sections of carotid arteries (injured and contralateral) for cryosection and histology was performed as described previously.^4^ Histological staining was performed using Accustain elastin stain (Sigma-Aldrich ACCUSTAIN; Egham UK), and areas of interest and circumferences were calculated using the image software Zeiss Axiovision (Zeiss). Lesions with neointima formation were encircled and the neointimal areas calculated. Medial area represented as the area between external elastic lamina (EEL) and internal elastic lamina (IEL) and the lesion area with neointima formation were calculated by subtracting lumen area from the internal elastic lamina area. Calculated neointimal area was then normalized to the medial area and expressed as intima media ratio. For visualization of T regulatory cells, 10 µm thick sections of spleen and carotid arteries (injured and contralateral) were fixed overnight in 4% formaldehyde in PBS and GFP-fluorescence was examined on a Zeiss LSM 5 laser scanning confocal microscope at 20× magnification. In the spleen, 1–2 images of the white pulp were obtained per section and 12 sections per mouse were inspected. The number of GFP-positive cells per fixed area (0.03 mm x mm) was calculated using the Zeiss LSM 5 analysis software. For carotid arteries, 6 sections per mouse were inspected. Consecutive sections to those used for confocal experiments were stained with Harris hematoxylin for visualization of tissue architecture.

**Figure 7:**
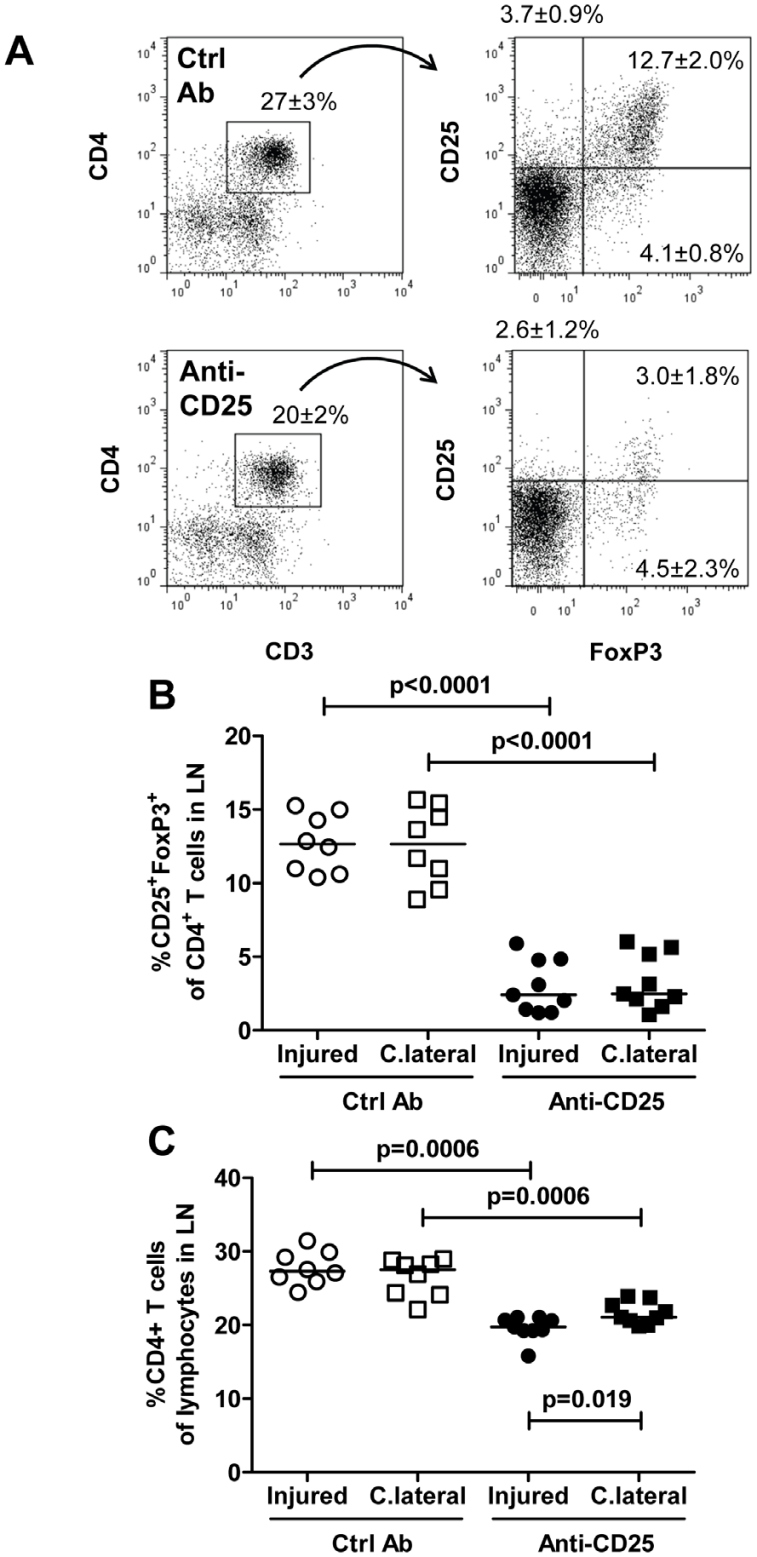
Reduced regulatory T cells in draining lymph nodes after injury of the carotid artery and treatment with anti-CD25. Cells were isolated from pooled lymph nodes (LN) of injured or sham-operated mice, stained with antibodies against CD3, CD4, FoxP3 and CD25 and analyzed by flow cytometry. A. Representative dot plots. B. FoxP3^+^ CD25^+^ cells as a percentage of CD3^+^CD4^+^ T cells and C. CD4^+^ T cells of lymphocytes in lymph nodes draining injured and uninjured contralateral carotid arteries. C.lateral, contralateral; Ctrl Ab, control antibody.

**Figure 8:**
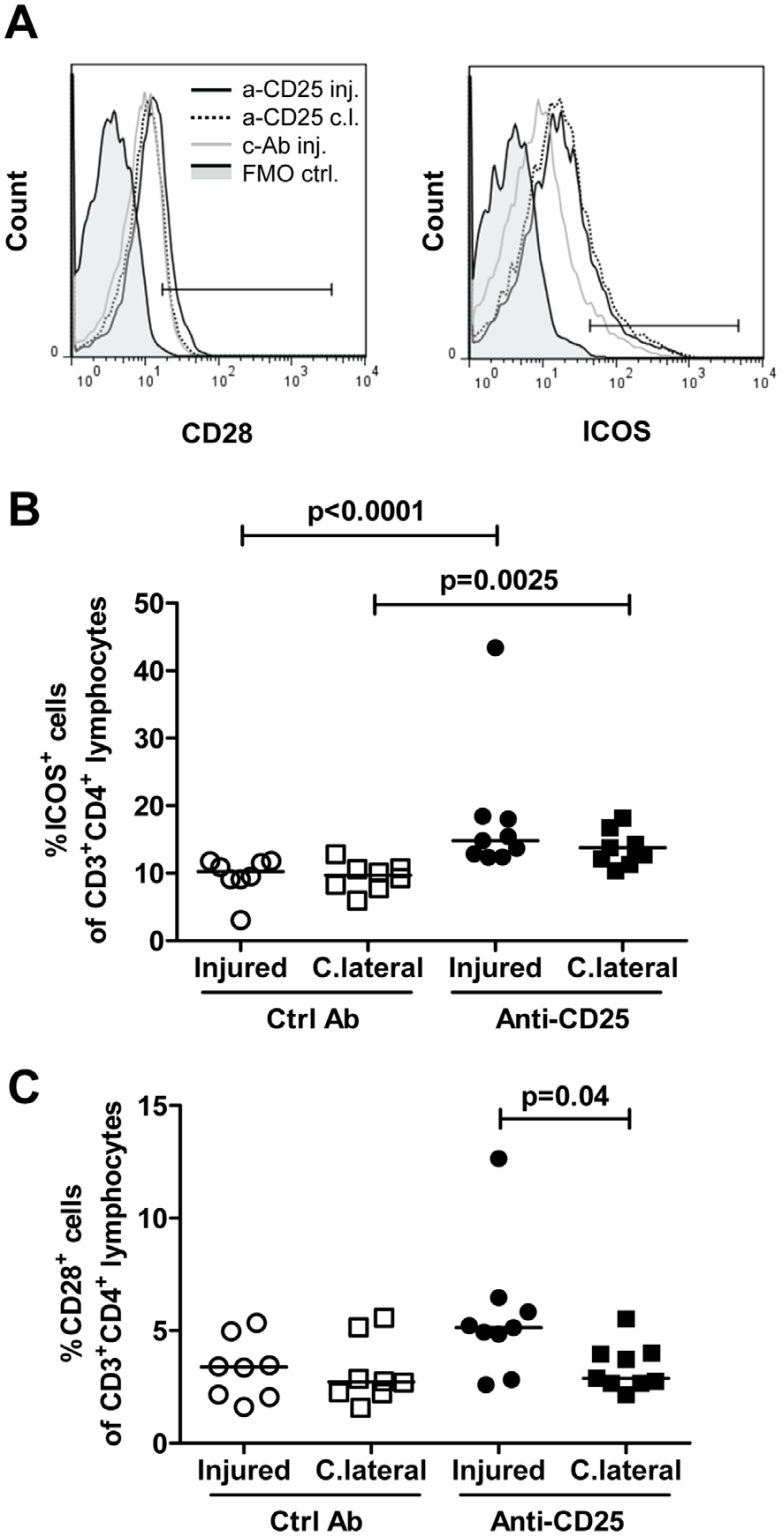
Increased CD28^+^ and ICOS^+^ T cells in draining lymph nodes after injury of the carotid artery and blockade with anti-CD25. Cells were isolated from draining lymph nodes of injured or uninjured contralateral carotid arteries, stained with antibodies against CD3, CD4, CD28 and ICOS and analyzed by flow cytometry. A. Representative histograms. Gate boundaries were set by fluorescence minus one controls (FMO ctrl, solid grey). B. ICOS^+^ cells as a percentage of CD3^+^CD4^+^ T cells. C. CD28^+^ cells as a percentage of CD3^+^CD4^+^ T cells. C.lateral and c.l., contralateral; Ctrl Ab and c-Ab, control antibody; inj, injured.

**Figure 9:**
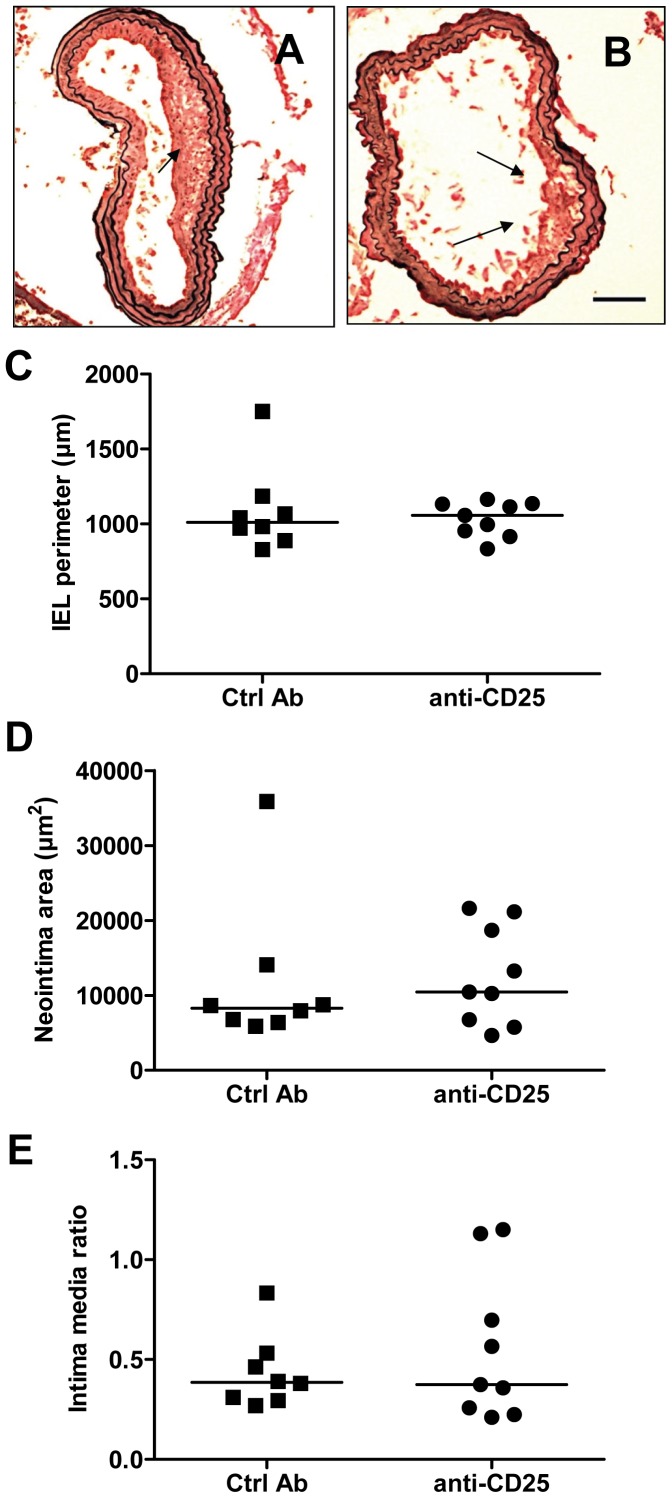
Deletion of regulatory T cell by anti-CD25 does not alter the vascular response to injury. Morphometric analysis of sections of injured carotid arteries. Photomicrograph of injured carotid artery section from mouse treated with A. Control antibody. B. Anti-CD25. Scale bar 100 µm. Arrows indicate neointimal thickenings. C. Perimeter of the internal elastic lamina (IEL). D. Area of neointima. E. Intima-media ratio. Ctrl Ab, control antibody.

### Flow Cytometry Analysis

Deep cervical lymph nodes and spleens were collected from female WT, Tap1^0^ and H2^0^ mice 3 days after collar injury. Cell suspensions were prepared by standard procedures, blocked with 2.4G2 mAb (anti-CD16/32 Fc block) and subsequently stained with various fluorochrome-conjugated antibodies and analyzed with flow cytometry on a CyAn ADP instrument (Beckman Coulter) as previously described [15]. Antibodies used in these experiments were phycoerythrin (PE)/cyanine-7 (Cy7)-anti-CD3e, Alexa Fluor (AF) 700-anti-CD4, allophycocyanin (APC)-anti-CD25, Pacific Blue (PB)-anti-FoxP3, fluorescein isothiocynate (FITC)-anti-CD28, PE/cyanine-5 (Cy5)-anti-ICOS, FITC-anti-IFNγ and Cascade Yellow-streptavidin biotin-anti-IL-4 (BioLegend).

### ELISA

Blood plasma was collected from mice at one day intervals for up to 7 days after surgical placement of collars around the right carotid arteries (2 mice per time point). The plasma levels of IL-1β and TNFα were measured with a MILLIPLEX MAP cytokine kit (Millipore, Billerica, MA, USA) on a Luminex LX100 instrument (Luminex corp., Austin, TX, USA) following the manufacturer’s protocol.

### Statistical Analysis

Statistical analysis was performed using GraphPad version 5 (GraphPad Software Inc., LaJolla, CA, USA). Values are presented as mean±SD, unless indicated. Analyses of distributions were performed before decisions were made to use parametric tests. For multiple comparisons, ANOVA or non-parametric Kruskal–Wallis tests were used to evaluate statistical significance. For comparisons between 2 groups, Student’s t-test or non-parametric Mann-Whitney tests were used. Results were considered statistically significant at P<0.05.

## Results

We first studied mobilization of CD4+ T cell subsets in draining lymph nodes 3 days after carotid collar injury. As compared with sham-operated mice there was a two-fold increase in the fraction of activated Th1 (IFNγ positive CD4^+^) T cells in lymph nodes from mice subjected to carotid collar injury (p = 0.004), whereas there was no difference in the fraction of activated Th2 (IL-4 positive CD4^+^) T cells (figure 1). Carotid injury was also associated with a significant increase in the fraction of CD4^+^ FoxP3^+^ Tregs in draining lymph nodes at 3 days as compared with sham injury (figure 2). To further characterize the role of Tregs in the carotid response to injury we performed collar injury in FoxP3-GFP transgenic C57/Bl6 mice. These mice express GFP under control of the *FoxP3* promotor and can be used to track FoxP3+ Tregs *in vivo*. No FoxP3-GFP+ cells were detected in the carotid artery wall of uninjured mice (day 0, figure 3). Three days after collar injury there was a substantial development of adventitial granulation tissue (figure 3). At this time point scattered FoxP3-GFP^+^ cells appeared in the granulation tissue while no cells were detected inside the carotid intima or media. At 7 days after injury FoxP3-GFP^+^ cells were still present in the adventitial granulation tissue but the intensity of the signal was weaker. The FoxP3-GFP^+^ cells appeared to have migrated closer to the medial layer but did not infiltrate the media. To determine the systemic Treg response to carotid injury we analyzed changes in the spleen population of FoxP3-GFP^+^ cells. The number of FoxP3-GFP^+^ cells in the spleen was found to be reduced by more than 75% 3 days after injury but had almost returned to the level of uninjured control mice after 7 days (figure 4). There was no difference in the fraction of IFNγ or IL-4 positive CD4^+^ T cells in the spleen between carotid and sham injured C57/Bl6 mice at 3 days after injury (2.0±1.5% versus 1.5±0.9% and 0.4±0.3% versus 0.3±0.0%, respectively). To further characterize the systemic response to carotid injury we analyzed circulating IL-1β levels during a one week period after injury. Plasma IL-1β levels increased progressively during the first 3 days after injury to subsequently decrease to the level of uninjured controls by day 6 (figure 5). In contrast, TNFα did not display a similar variation over days after injury (figure 5).

Since our findings suggest local involvement of several different CD4^+^ T cell subtypes we next performed carotid collar injury in mice lacking expression of MHC class II molecules (H2^0^ mice), mice which are therefore unable to activate CD4+ T cells. As a consequence of the MHC class II deficiency these mice also have severely reduced levels of effector and regulatory CD4^+^ T. Despite this reduction, no difference in neointima formation or intima/media ratio was observed between MHC class II-deficient and WT mice at 21 days (figure 6). Recent studies have reported that transfer of CD8^+^ T cells into Rag-1 mice lacking functional T and B cells reduces neointima formation after carotid injury. To study the role of MHC class I-dependent activation of CD8^+^ T cells in presence of functional CD4^+^ T cells and B cells we performed carotid collar injury in mice deficient in the transport of class I (Tap1) protein. This protein is required for presentation of antigens on MHC class I. Tap1 deficiency is associated with an almost complete loss of MHC class I surface expression as well as of CD8^+^ T cells. However, we found no difference in neointima formation or intima/media ratio between wild type and Tap1-deficient mice (figure 6).

It is known that CD4^+^ Th1 cells and Tregs have opposing effects on activation of inflammation, CD4^+^ Th1 cells being pro-inflammatory whereas Tregs have immune-suppressive and anti-inflammatory actions. Accordingly, it is possible that the simultaneous activation of both of these T cell subsets in response to arterial injury also generates opposing responses in respect to the modulation of neointima formation. We therefore performed new experiments in which Tregs were partially removed through injection of CD25- blocking antibodies. Treatment with CD25 antibodies reduced the fraction of CD4^+^CD25^+^FoxP3^+^ Tregs both in draining and contralateral lymph nodes by more than 80% (figure 7). Anti-CD25-treatment was also associated with an increased expression of the T cell activation marker ICOS on CD4^+^ T cells in both draining and contralateral lymph nodes confirming that the reduction of Tregs affected CD4^+^ T cell activation (figure 7). CD25 antibody treatment also resulted in an increased expression of the activation marker CD28 on CD4+ T cells in draining lymph nodes after injury as compared to contralateral lymph nodes (figure 8). However, there was no significant difference in neointima formation or intima/media ratio between mice treated with CD25 antibodies and mice given an irrelevant control antibody (figure 9).

## Discussion

T cells have been attributed an important role in modulating repair responses following vascular injury but the role of different T cell subsets remains to be fully understood [1]. The present study demonstrates that carotid injury is associated with an early (day 3) mobilization of bothTh1 T cells and CD4^+^CD25^+^FoxP3^+^ Tregs in draining lymph nodes. Our data also suggest that carotid injury is associated with an emigration of Tregs from the spleen and that 3 days after carotid injury less than 25% of the original Treg population remain in the spleen. Tregs did not accumulate in the intima or media of the injured artery itself but were observed scattered in the adventitial granulation tissue.

Th1 T cells have indirectly been implicated in the modulation of neointima formation after injury through their release of IFNγ, a potent inhibitor of smooth muscle cell proliferation. Accordingly, treatment with IFNγ has been shown to reduce neointima formation, as well as the intimal proliferation of smooth muscle cells, following carotid balloon catheter injury in rats [3]. Studies by Dimayuga and coworkers suggest that the role of IFNγ in modulating neointima formation is bimodal with an early inhibitory effect followed by a later stimulatory effect [13]. To study the net effect of CD4^+^ T cells on neointima formation in response to carotid injury we compared wild type and MHC class II deficient mice. The latter are unable to present antigens to CD4^+^ T cells and are also characterized by dramatic reduction of both CD4^+^ effector and regulatory T cells. The observation that there was no difference in neointima formation between wild type and MHC class II deficient mice suggest that CD4^+^ T cells either are not involved in modulating the repair process or that different CD4^+^ T cell subtypes have counter-active effects. This finding is in line with previous studies demonstrating that transfer of CD4^+^ T cells does not influence neointima formation in Rag-1^−/−^ mice [14]. However, in this context it is important to note that MHC class II deficient mice have a compensatory increase in CD8^+^CD25^+^ T cells that share phenotypic and functional properties with regulatory CD4^+^CD25^+^FoxP3^+^ T cells [16] and that it is difficult to exclude that these cells may have influenced the outcome of the present study.

The activation of Tregs in response to arterial injury has not been previously described and their role in modulating the subsequent repair process is unknown. As Tregs are known to counteract the effect of Th1 it is possible that they may increase neointima formation by inhibiting IFNγ producing Th1 cells. Accordingly, it is possible that arterial injury leads to the activation of CD4^+^ T cells with partially opposing effect on neointima formation and that the lack of effect observed in MHC class II deficient mice is explained by a loss of all of these cells. To determine if a possible effect of Th1 cells on neointima formation was counteracted by a parallel activation of Tregs we treated wild type mice with CD25 blocking antibodies, a well-established approach for deletion of Tregs [17]. Although this treatment was associated with a 80% reduction of Tregs in both draining and contralateral lymph nodes, as well as with signs of increased activation of remaining CD4^+^ T cells, it did not influence neointima formation suggesting that neither Tregs nor Th1 T cells influence vascular repair responses in this model. However, our findings need to be interpreted with some caution since it cannot be completely excluded that CD25 antibody treatment also deleted some CD25-expressing CD4^+^ T effector cells. Moreover, it is also possible that Tregs themselves may have dual effects on neointima formation in the same way as IFNγ producing Th1 cells [13]. Treatment with CD25 antibodies increased the expression of ICOS on remaining CD4^+^ T cells. Transfer of ICOS^−/−^ bone marrow has previously been shown to aggravate atherosclerosis in LDL receptor deficient mice suggesting a protective role of this co-stimulatory molecule [18]. The possible role of ICOS in repair responses to arterial injury is unknown but the present findings demonstrate that an up-regulation of ICOS on CD4^+^ T cells does not appear to influence neointima formation.

An interesting and unexpected observation was that carotid injury was associated with a dramatic emigration of Tregs from the spleen. Three days after injury only 25% of the original Treg population remained in the spleen. The splenic emigration of Tregs appeared to be temporary because at day 7 day after injury Treg numbers were almost back to the same level as in uninjured mice. Swirski and coworkers [19] recently reported that splenic pro-inflammatory monocytes in response to ischemic myocardial injury exit the spleen en masse, accumulate in injured tissue, and participate in wound healing. It is likely that the emigration of Tregs in response to carotid injury observed here represents a parallel regulatory response aimed to control inflammation in the injured tissue. We have previously shown that carotid injury is associated with a local accumulation of monocytes and that these frequently localize to the periadventitial tissue where they promote neointima formation through release of IL-1β [4]. Carotid collar injury is associated with a rapid formation of adventitial granulation tissue (figure 3). It is an interesting possibility that the primary function of the Tregs recruited in response to collar injury is to control inflammation in this tissue rather than the subsequent neointima formation.

The finding of increased neointima formation in both T cell-depleted and *Rag-1* ^−/−^ mice suggest the existence of a T cell population with inhibitory properties [12]. Dimayuga and coworkers recently reported that transfer of CD8^+^ T cells inhibits neointima formation in *Rag-1* ^−/−^ mice and provided evidence that this effect may be mediated through a cytotoxic activity against intimal smooth muscle cells [14]. In the present study we analyzed the role of CD8^+^ T cells in neointima formation in Tap1^0^ mice that have severely reduced MHC class I expression and number of CD8^+^ T cells. However, in contrast to the studies by Dimayuga and coworkers we did not observe any effect on neointima formation. The reasons for the different outcomes remain to be clarified but may involve differences in the models used. *Rag-1* ^−/−^ mice are completely devoid of functional T and B cells, whereas Tap1^0^ mice have both functional CD4^+^ T cells and B cells. Since B cells previously has been shown to reduce neointima formation in *Rag-1* ^−/−^ mice [12] it is possible that the B cells present in Tap1^0^ mice are sufficient to suppress any enhanced neointima formation due to lack of CD8^+^ T cells in these mice. It should also be kept in mind that although the CD8^+^ T cells constitute less than 1% of the total lymphocyte population in Tap1^0^ mice [20], these mice still have the ability to generate a small population of functionally intact CD8^+^ T cells [21] that may have affected neointima formation in our studies.

In conclusion, the present observations demonstrate that carotid injury is associated with pro-inflammatory responses, such as activation of CD4^+^IFNγ^+^ Th1 cells and IL-1β release, but also mobilization of potentially anti-inflammatory CD4^+^CD25^+^FoxP3^+^ Tregs. Depletion of Tregs did not, however, influence the subsequent repair processes leading to the formation of a neointima. They also demonstrate that lack of CD8^+^ T cells does not influence neointima formation in the presence of functional CD4^+^ T cells and B cells.
